# Laying the foundations for patient‐centered research in twin‐to‐twin transfusion syndrome

**DOI:** 10.1002/pmf2.70195

**Published:** 2025-12-19

**Authors:** Jessian L. Munoz, Alireza A Shamshirsaz

**Affiliations:** ^1^ Department of Obstetrics and Gynecology Baylor College of Medicine and Texas Children's Fetal Center Houston Texas USA; ^2^ Fetal Care and Surgery Center Boston Children's Hospital Harvard Medical School Boston Massachusetts USA

Dear Editor,

We appreciate the thoughtful letter from Drs Paek and Miller regarding our editorial “TTTS Staging Updates; Balancing Evidence, Access and Patient Care.” In addition, we value and acknowledge their contributions through the Delphi consensus study, which represents an important step toward harmonizing the terminology and clinical management of twin‐to‐twin transfusion syndrome (TTTS), particularly to the less studied earlier stage or those deemed “atypical” presentations.

We agree that increased vigilance in the setting of amniotic fluid discordance, called “pre‐TTTS” in the Delphi study, represents a valuable opportunity for early characterization, detection, and potential prevention of disease progression. The proposed emphasis on shorter intervals and Doppler assessment, specifically of the umbilical vein and ductus venous, while aligned with the current SMFM recommendations, is best utilized for a goal for timely intervention while minimizing unnecessary referral, which could impact patient access and increased burden. Prospective assessment of this approach to triaging should be studied with an emphasis on access and overall healthcare cost.

The distinction between “pre‐TTTS,” “atypical TTTS,” and current established staging of TTTS represents an important conceptual advancement, which highlights the spectrum of the disease, the nuances in hemodynamic evaluations, and the true progress of understanding pathophysiology in our growing field. As such, it can be appreciated that the authors have clarified that “atypical TTTS” involves both cardiac compromise and polyhydramnios of the recipient twin, even in the absence of donor twin oligohydramnios, but we agree that echocardiographic evaluation should not be required for diagnosis given the variability in access to advanced cardiac imaging. Further studies on the optimal management of these “atypical” twins are needed to assess for optimal treatment approaches and outcomes, which likely will differ from presentations that are more classical [1].

Lastly, advocacy for integrated models of telemedicine in TTTS management is commendable. This collaborative approach with regional screening networks, supported by the fetal therapy centers, can have a significant impact by reducing geographic and financial barriers while maintaining diagnostic rigor and safety. These models have been effective for complex placental pathologies and maternal mental health assessments [2, 3]. The mode described is worthy of broader implementation likely to enhance patient and provider satisfaction.

In summary, we share the authors’ vision of harmonizing accuracy, surveillance, and accessibility to ensure optional outcomes for all families affected by complications of monochromic twin pregnancies. The Delphi‐based recommendations may serve as a foundation for the next phase of TTTS care, collaboration, and research.

## CONFLICT OF INTEREST

None of the authors have a conflict of interest to disclose.

## FUNDING INFORMATION

The authors received no specific funding for this work.

## Data Availability

Data sharing is not applicable to this article as no datasets were generated or analyzed during the current study.
